# Rab27a plays a dual role in metastatic propensity of pancreatic cancer

**DOI:** 10.1038/s41598-020-64248-1

**Published:** 2020-04-30

**Authors:** Nancy Kren, Daniel Michaud, Sukriti Bagchi, Kevin Greene, Yuliya Pylayeva-Gupta

**Affiliations:** 1 Lineberger Comprehensive Cancer Center, The University of North Carolina at Chapel Hill, School of Medicine, Chapel Hill, North Carolina USA; 2 Department of Cell Biology and Physiology, The University of North Carolina at Chapel Hill School of Medicine, Chapel Hill, North Carolina USA; 3 Department of Pathology and Laboratory Medicine, The University of North Carolina at Chapel Hill School of Medicine, Chapel Hill, North Carolina USA; 4 Department of Genetics, The University of North Carolina at Chapel Hill School of Medicine, Chapel Hill, North Carolina USA; 5 Present Address: University of Arizona, AZ, USA

**Keywords:** Pancreatic cancer, Metastasis

## Abstract

Pancreatic cancer is an aggressive malignancy, often diagnosed at metastatic stages. Several studies have implicated systemic factors, such as extracellular vesicle release and myeloid cell expansion, in the establishment of pre-metastatic niches in cancer. The Rab27a GTPase is overexpressed in advanced cancers, can regulate vesicle trafficking, and has been previously linked to non-cell autonomous control of tumor growth and metastasis, however, the role of Rab27a itself in the metastatic propensity of pancreatic cancer is not well understood. Here, we have established a model to study how Rab27a directs formation of the pre-metastatic niche. Loss of Rab27a in pancreatic cancer cells did not decrease tumor growth *in vivo*, but resulted in altered systemic myeloid cell expansion, both in the primary tumors and at the distant organ sites. In metastasis assays, loss of Rab27a expression in tumor cells injected into circulation compromised efficient outgrowth of metastatic lesions. However, Rab27a knockdown cells had an unexpected advantage at initial steps of metastatic seeding, suggesting that Rab27a may alter cell-autonomous invasive properties of the tumor cells. Gene expression analysis of gene expression revealed that downregulation of Rab27a increased expression of genes involved in epithelial-to-mesenchymal transition pathways, consistent with our findings that primary tumors arising from Rab27a knockdown cells were more invasive. Overall, these data reveal that Rab27a can play divergent roles in regulating pro-metastatic propensity of pancreatic cancer cells: by generating pro-metastatic environment at the distant organ sites, and by suppressing invasive properties of the cancer cells.

## INTRODUCTION

Over 90% of pancreatic intraepithelial neoplasia (PanIN) lesions contain oncogenic mutations in the Kras locus, making it the most common and likely the initiating transformation event in pancreatic ductal adenocarcinoma (PDA)^1^. The field of pancreatic cancer biology has benefited tremendously from the development of spontaneous mouse models of pancreatic carcinogenesis, which feature pancreas-specific endogenous expression of oncogenic Kras and the tumor suppressor p53, and faithfully recapitulate formation and histopathological progression of PDA to metastasis^2,3^. Importantly, both human and mouse PanINs feature extensive remodeling of surrounding microenvironment characterized by pronounced influx of immune cells, which has been shown to shape inflammatory and immunosuppressive pro-tumorigenic milieu^4–6^. While it is appreciated that host immunity plays a critical role in regulating tumorigenesis, the mechanisms behind immune response in pancreatic cancer are poorly understood. Furthermore, systemic immune changes have been documented in patients with metastatic pancreatic cancer, which most commonly metastasizes to liver, lung and diaphragm, however, our understanding of the role of the immune system in the process of pancreatic cancer metastasis remains scarce.

Systemic factors released by primary tumors have been shown to influence metastasis via a variety of mechanisms. For example, in models of lung cancer and melanoma it has been shown that secretion of VEGF and PIGF from the primary tumor increase levels of MMP9 in the lung and facilitate distant metastasis^7^. Accumulation of hematopoietic cells at distant sites have also been shown to facilitate metastasis, for example release of inflammatory proteins such as S100A8 results in accumulation of activated macrophages in the lung^8^. MicroRNAs have also been shown to promote metastasis. In a breast cancer model it was shown that miRNA 105 released from tumors contributed to the breakdown of vascular endothelial barriers by targeting ZO1^9^. Extracellular vesicles (EV) can be released by cancer cells and have been postulated to influence a variety of pro-metastatic properties in part by altering immunological milieu of a variety of distant organ sites in systemic fashion^10,11^. Extracellular vesicles have been implicated in many functions that regulate tumor progression, including immune escape, invasion and metastasis^11–14^. In pancreatic cancer, ectopic transfer of EVs promoted the recruitment and activation of myeloid cells, potentiated establishment of pre-metastatic niche and experimental metastases^11,15^. Rab27a is a small Ras-like GTPase that has been shown to be differentially expressed in cancer and is known to contribute to EV biogenesis^10,16,17^. Overexpression of Rab27a has been linked to poor prognosis in cancer, presumably in the context of the contribution to the extensive remodeling by systemic EV release^12,18^. However, the role of Rab27a itself in the metastatic propensity of pancreatic cancer *in vivo* remains unclear. In this study, we aimed to address the role of Rab27a in modulating metastatic properties of pancreatic cancer *in vivo*.

## Results

### Primary pancreatic tumors promote expansion of myeloid cell subsets at distant organ sites

**Figure 1:**
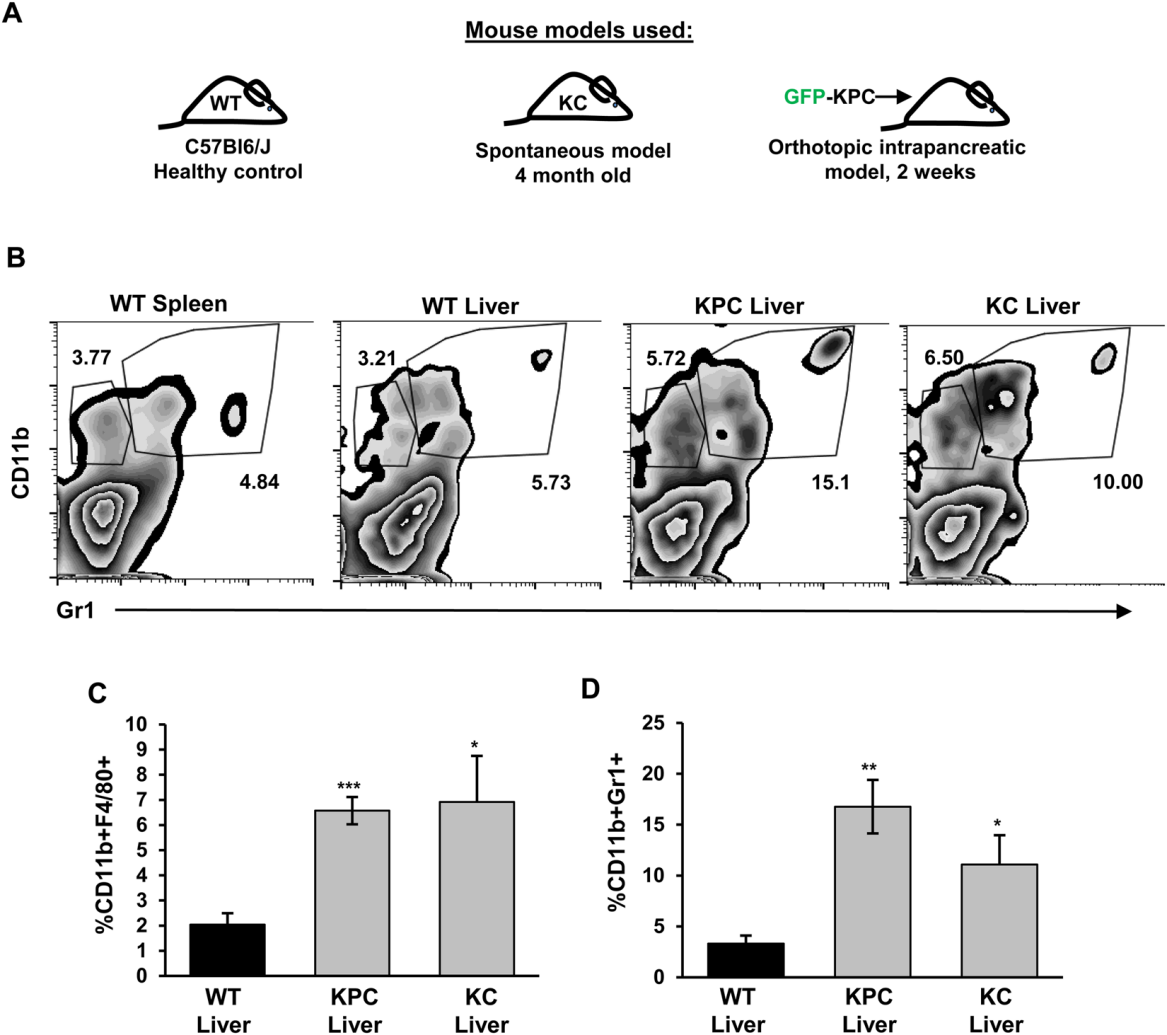
Myeloid cell subsets accumulate in distant organs of mice with primary pancreatic tumors. **(A)** Schematic of mouse models used in Fig. 1. **(B)** Representative flow cytometry analysis of CD45^+^CD11b^+^Gr1^+/-^ myeloid cells in spleen and livers of WT, *KC* and *KPC* mice. Proportion of CD45^+^ cells is indicated. **(C)** Quantification of CD45^+^CD11b^+^Gr1^-^ myeloid cell frequency in spleen and livers of WT, *KC* and *KPC* mice. Proportion of CD45^+^ cells is indicated. **(D)** Quantification of CD45^+^CD11b^+^Gr1^+^ myeloid cell frequency in spleen and livers of WT, *KC* and *KPC* mice. Proportion of CD45^+^ cells is indicated. Error bars indicate SEM; p value: *<0.05; **<0.01; ***<0.001. Data represents 3 independent experiments.

To understand how the immunological milieu at distant organ sites is affected by the presence of primary pancreatic tumors, we used a well-established Kras-driven model of spontaneous pancreatic tumorigenesis, *LSL-Kras*^*G12D/+*^*; p48*^*Cre/+*^ (*KC*), which is not proficient at driving metastasis^2^. To identify immune infiltrates in livers of mice with primary tumors, livers from WT and *KC* mice were enzymatically digested and processed for a flow cytometry-based isolation of liver-associated immune cell populations. Consistent with published findings, we detected significant increases in the frequency of myeloid cells, including CD11b^+^Gr1^+^ and CD11b^+^F4/80^+^ subsets, in the livers of 4 month-old *KC* animals as compared to control WT mice (Fig. 1A–D)^6,11^.

To understand if changes in the immune microenvironment of the liver can be recapitulated in a tractable metastatic model of pancreatic cancer, we orthotopically injected GFP-labeled cancer cells that were derived from a *LSL-Kras*^*G12D/+*^*; LSL-Trp53*^*R172H/+*^*; p48*^*Cre/+*^ mouse model (*GFP-KPC* cells) into the pancreata of syngeneic WT mice^3,19^. At 2 weeks post-implantation, we observed significant expansion of both CD11b^+^F4/80^+^ and CD11b^+^Gr1^+^ myeloid cells in both the pancreata and livers of animals with primary *KPC* tumors (Fig. 1A–D), which was in alignment with our findings in an autochthonous model. We also observed a small, but significant increase in CD4^+^ T cells in the livers of *KC* mice, but not in *KPC* mice (Supplementary Figure 1A,B). We observed no significant changes in CD8^+^ T cells, γδ T cells, NK cells, or dendritic cells in the livers of *KC* or *KPC* mice (Supplementary Figure 1A, C and D). Since pancreatic cancer cells in spontaneous models have been shown to disseminate to livers, it is possible that cancer cells that migrated from the primary tumor site to liver could contribute to expansion of myeloid cells^3,20^. While we observed a sizable tumor lesion in the pancreas at 2 weeks post-*KPC* cell injection, we did not detect *GFP-KPC* cells in the livers of animals at this early timepoint (Supplementary Figure 1E), suggesting that most immunological changes in livers at this timepoint are likely to reflect the presence of systemic factors provided by the primary tumor milieu.

We also wanted to ask if the nature of the systemic signal that elicits immune changes in organs other than pancreas is restricted to the liver. To that end, we used WT mice or animals with *KPC* primary pancreatic tumors and profiled mouse lungs, the second most common site for pancreatic metastases, for changes in myeloid cell composition. We observed that, similar to the liver, lungs from mice with KPC tumors had significantly more myeloid cells than their WT counterparts (Supplementary Figure 1F). Specifically, the myeloid subsets CD11b^+^Gr1^+^ were significantly enriched, while frequencies of macrophages remained unchanged as compared to control mouse lungs (Supplementary Figure 1G, H) suggesting potential differences in systemic reprogramming of distant niches.

### **Primary pancreatic cancer facilitates metastatic seeding**

**Figure 2:**
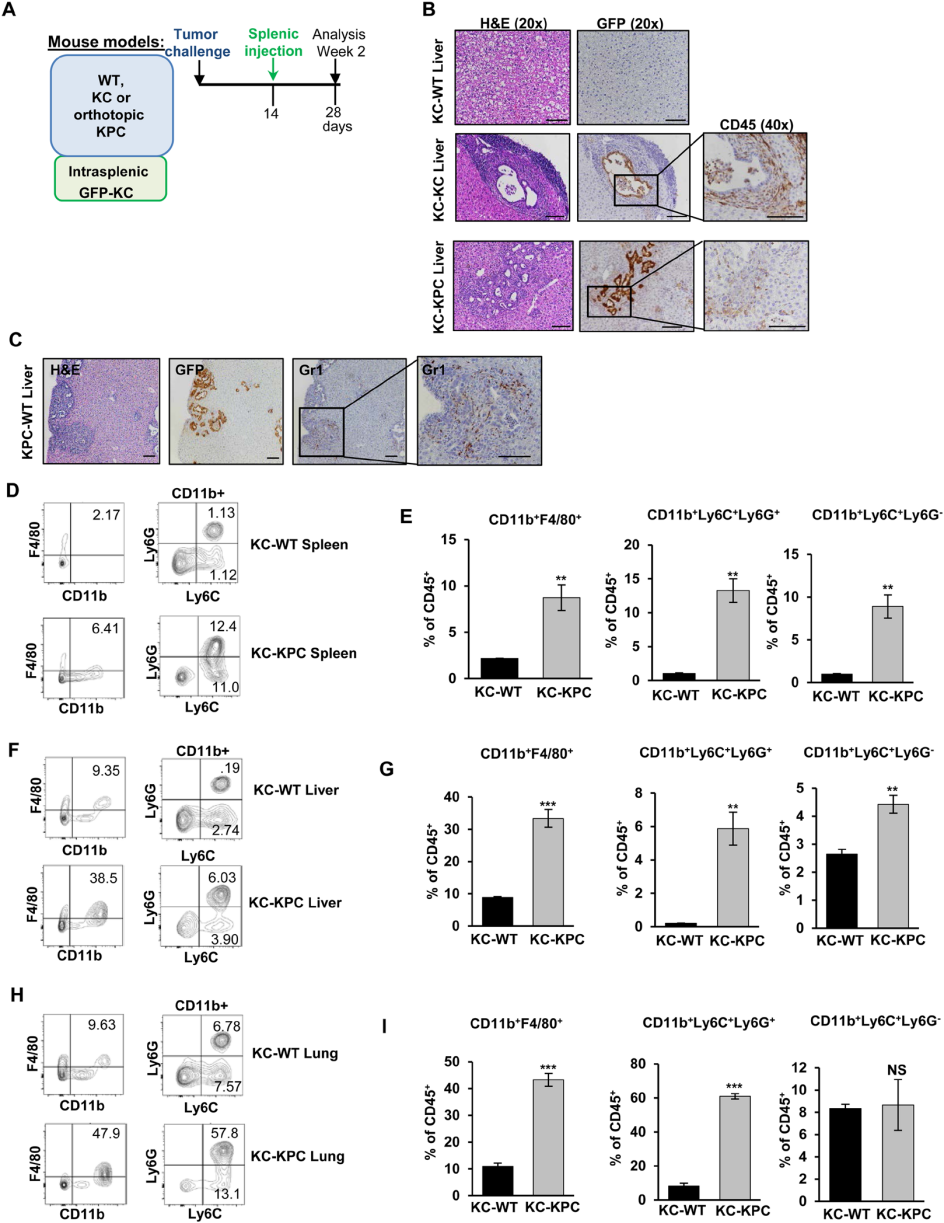
Primary pancreatic tumors facilitate metastatic seeding. **(A)** Schematic of mouse models used in Fig. 2. **(B)** Representative histological images of livers of WT, *KC* or *KPC* mice intra-splenically injected with *GFP-KC* cells at 2 weeks post-injection, H&E, GFP and CD45 as labeled. 20x objective, scale bar represents 100um. **(C)** Representative histological images of livers of WT mice intra-splenically injected with *GFP-KPC* cells at 2 weeks post-injection. H&E, GFP, and Gr1 at 10×. Final panel represents inset of Gr1 at 20×. Error bars represent 100um. **(D)** Representative flow cytometry plots of CD45^+^CD11b^+^F4/80^+^ and CD45^+^CD11b^+^Ly6G/Ly6C^+^ cells from the spleens of WT or *KPC* mice intra-splenically injected with *GFP-KC* cells at 2 weeks post-injection. **(E)** Quantification of CD45^+^CD11b^+^F4/80^+^ and CD45^+^CD11b^+^Ly6G/Ly6C^+^ cells from the spleens of WT or *KPC* mice intra-splenically injected with *GFP-KC* cells at 2 weeks post-injection. **(F)** Representative flow cytometry plots of CD45^+^CD11b^+^F4/80^+^ and CD45^+^CD11b^+^Ly6G/Ly6C^+^ cells from the livers of WT or *KPC* mice intra-splenically injected with *GFP-KC* cells at 2 weeks post-injection. **(G)** Quantification of CD45^+^CD11b^+^F4/80^+^ and CD45^+^CD11b^+^Ly6G/Ly6C^+^ cells from the livers of WT or *KPC* mice intra-splenically injected with *GFP-KC* cells at 2 weeks post-injection. **(H)** Representative flow cytometry plots of CD45^+^CD11b^+^F4/80^+^ and CD45^+^CD11b^+^Ly6G/Ly6C^+^ cells from the lungs of WT or *KPC* mice intra-splenically injected with *GFP-KC* cells at 2 weeks post-injection. **(I)** Quantification of CD45^+^CD11b^+^F4/80^+^ and CD45^+^CD11b^+^Ly6G/Ly6C^+^ cells from the lungs of WT or *KPC* mice intra-splenically injected with *GFP-KC* cells at 2 weeks post-injection. Error bars indicate SEM; NS – not significant, p value: **<0.01; ***<0.001. Data represents 3 independent experiments.

Next, we set out to determine whether the presence of primary pancreatic lesions may facilitate metastatic outgrowth. To test this idea, we injected non-metastatic *GFP-KC* cells into the spleens of either wild-type mice (*KC-WT*), mice with spontaneous *KC* primary neoplasia (*KC-KC*) or mice with primary orthotopic *KPC* tumors (*KC-KPC*) (Fig. 2A). Liver tissues were harvested at 4 weeks post-injection and analyzed for the presence of GFP^+^ cells. *GFP-KC* cells injected into spleens of WT animals were not detectable at this timepoint (Fig. 2B). Microscopic analysis of livers of *KC-KC* or *KC-KPC* mice revealed the presence of *GFP-KC* ductal lesions (Fig. 2B). Although infrequent, these lesions show significant accumulation of CD45^+^ immune cells (Fig. 2B). In contrast, injection of more metastatic KPC cells into spleens of WT mice (*KPC-WT*) revealed a robust formation of metastases surrounded by Gr1^+^ myeloid cells (Fig. 2C). These results support the idea that the altered microenvironment in the livers of mice with pancreatic cancer may better support the growth of cancer cells, even if the cells themselves are not overtly metastatic. Concordant with our observations in the setting of *KC* and/or KPC primary tumors, we observed significant increases in the frequencies of macrophages and CD11b^+^Gr1^+^ myeloid subsets in spleens, livers and lungs of *KC-KPC* animals with primary *KPC* tumors, whereas administration of *GFP-KC* cells into naïve livers alone (*KC-WT*) was not sufficient to alter immunological milieu (Fig. 2D–I). The metastatic events by *KC* cells injected intrasplenically, however, were not efficient enough to provide robust system for quantifying metastatic outgrowth. For these reasons the remainder of our studies were conducted with *KPC* cells, as they form much more readily quantifiable metastases.

### **Knockdown of Rab27a affects*in vitro*, but not*in vivo*cancer cell growth**

**Figure 3:**
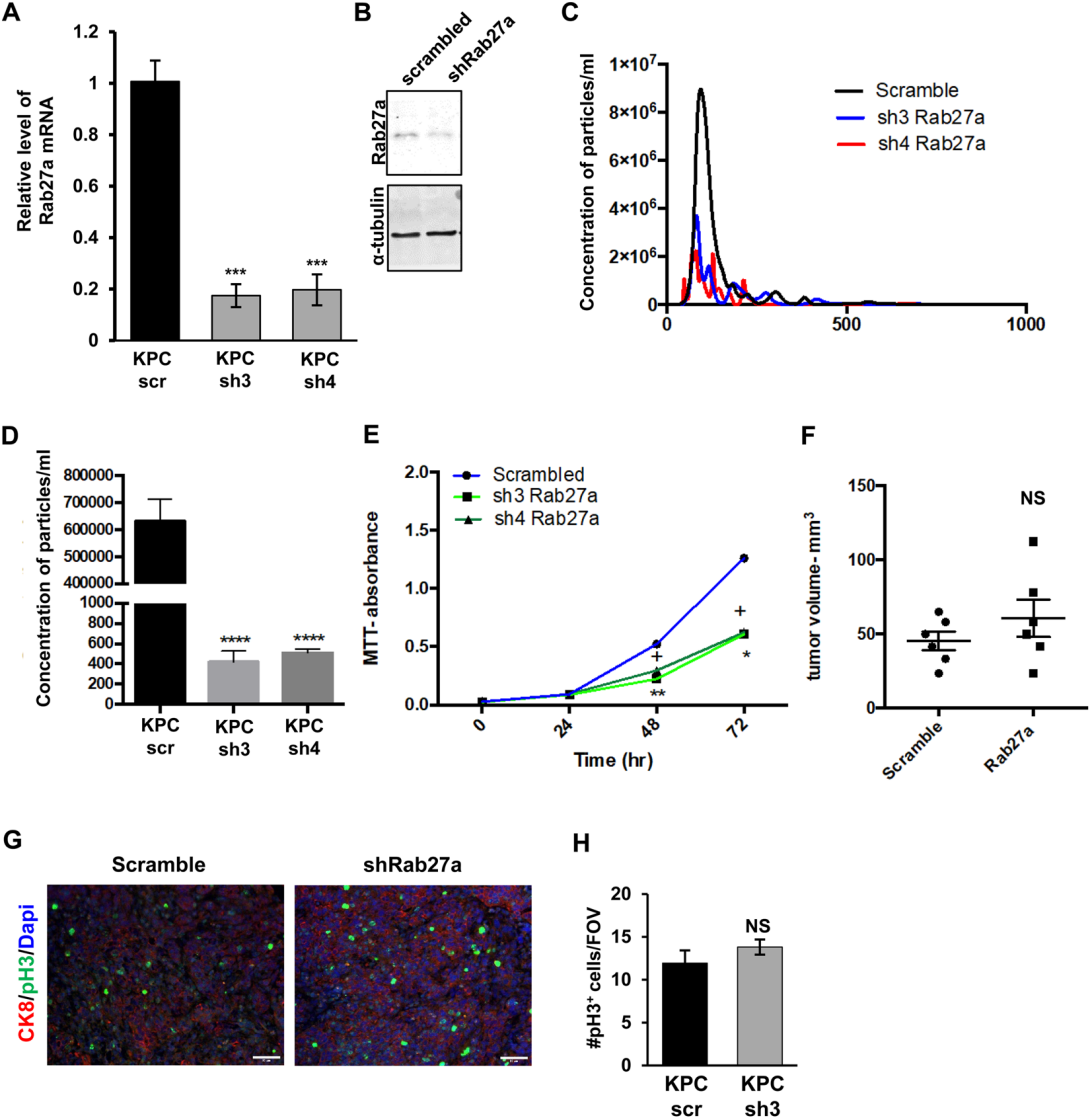
Knockdown of *Rab27a* in *KPC* cells decreases EV release, but does not alter primary tumor growth *in vivo*. **(A)** Levels of *Rab27a* mRNA in *KPC* cells were assessed by quantitative RT-PCR. **(B)** Levels of *Rab27a* protein in *KPC* cells were assessed by Western blotting. **(C)** Nanosight profile of vesicle distribution in supernatants collected from *KPC* cells infected with scrambled vector or shRNA constructs directed against *Rab27a*. **(D)** Nanosight particle concentration of supernatants collected from *KPC* cells infected with scrambled vector or shRNA constructs directed against *Rab27a*. **(E)** Growth curve for *Rab27a* knockdown *KPC* cells as compared to *Scrambled* control *KPC* cells. “+” indicated statistical difference between sh1 and scrambled control; “*” indicates statistical difference between sh2 and scrambled control. **(F)** Tumor volume at two weeks post injection of *KPC* cells infected with scrambled vector or shRNA constructs directed against *Rab27a*. **(G)** Phospho-Histone 3 immunofluorescent staining of tumors generated from *KPC* cells infected with scrambled vector or shRNA constructs directed against *Rab27a*. **(H)** Quantification of Phospho-Histone 3 immunofluorescent staining from (G). Error bars indicate SEM; NS – not significant, p value: *<0.05; ***<0.001. Data represents 3 independent experiments.

Modulation of the pre-metastatic niche microenvironment in pancreatic cancer has been linked to systemic production of factors, such as VEGF, PIFG, MMP9, and extracellular vesicles among others^7,11^. In this study, we wanted to understand the role of Rab27a in facilitating the ability of pancreatic cancer cells to alter distant niches and/or to affect local tumor outgrowth^10,17^. Rab27a directs production of EVs, including exosomes, which have been postulated to facilitate myeloid cell accumulation, pre-metastatic niche formation, and/or metastatic outgrowth in a variety of cancers including pancreatic^11^. Therefore, we assessed whether we could efficiently decrease the release of EVs using knock-down of Rab27a as a strategy to modulate distant pre-metastatic niche. To do this, we knocked-down *Rab27a* expression in *KPC* cells using two separate shRNA constructs and validated significantly decreased expression at the RNA and protein levels (Fig. 3A-B and Supplementary Figure 2). Briefly, EVs were collected from the supernatant of *scr*-*KPC* or *shRab27a*-*KPC* cells and isolated by polymer precipitation. The size and quantity of the collected particles was determined by nanoparticle tracking analysis using the Nanosight NS500 (particle counts were normalized to cell number) (Fig. 3C,D). Figure 3C shows particle distribution for scrambled and two Rab27a hairpins and Fig. 3D depicts the average concentration of particles. To understand if reduction in levels of Rab27a led to any cell-autonomous changes, we measured the rate of cell growth of *scr*-*KPC* and *shRab27a-KPC* using an MTT proliferation assay. We observed a small, but reproducible decrease in the growth of *shRab27a-KPC* cells (Fig. 3E), consistent with previously published observations^21^. To understand if modulation of Rab27a expression also affects primary tumor growth, we orthotopically injected *scr*-*KPC* and *shRab27a*-*KPC* cells and measured tumor growth 2 weeks after injection. Despite a change in *shRab27a*-*KPC* cell growth *in vitro*, we did not observe significant differences in tumor volumes between control *scr*-KPC and *shRab27a*-KPC tumors (Fig. 3F). To further validate that tumor cell growth was not affected *in vivo*, we also checked the rate of proliferation in *scr*-*KPC* and *shRab27a*-*KPC* tumors by analyzing phospho-Histone H3 staining, and observed no changes (Fig. 3G, H). Overall this data suggests that slight *in vitro* modulation of cell growth by Rab27a in pancreatic cancer cells is not sufficient to alter *in vivo* growth rates.

### **Loss of Rab27a in primary tumors modulates distant immunological milieu**

**Figure 4:**
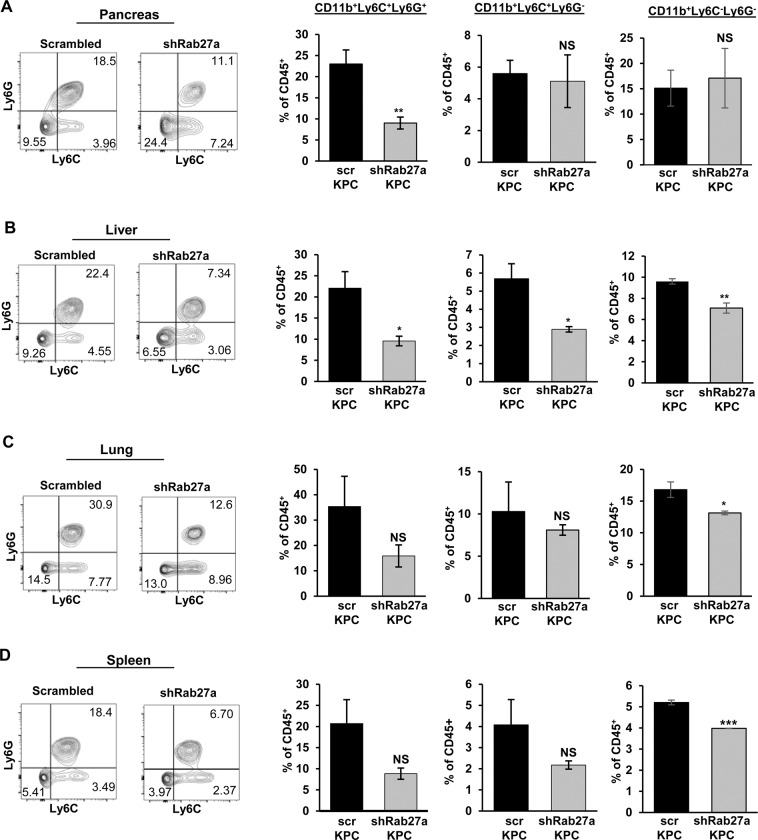
Knockdown of *Rab27a* in primary tumors has differential effect on systemic distribution of myeloid cell subsets. **(A)** Representative flow cytometry plots and quantification of CD45^+^CD11b^+^Gr1^-^ and CD45^+^CD11b^+^Ly6C/G^+^ myeloid cells in pancreata of WT mice orthotopically injected with *scr-KPC* or *shRab27a-KPC* cells, at 2 weeks post-injection. Proportion of CD45^+^ cells is indicated. **(B)** Representative flow cytometry plots and quantification of CD45^+^CD11b^+^Gr1^-^ and CD45^+^CD11b^+^Ly6C/G^+^ myeloid cells in livers of WT mice orthotopically injected with *scr-KPC* or *shRab27a-KPC* cells, at 2 weeks post-injection. Proportion of CD45^+^ cells is indicated. **(C)** Representative flow cytometry plots and quantification of CD45^+^CD11b^+^Gr1^-^ and CD45^+^CD11b^+^Ly6C/G^+^ myeloid cells in lungs of WT mice orthotopically injected with *scr-KPC* or *shRab27a-KPC* cells, at 2 weeks post-injection. Proportion of CD45^+^ cells is indicated. **(D)** Representative flow cytometry plots and quantification of CD45^+^CD11b^+^Gr1^-^ and CD45^+^CD11b^+^Ly6C/G^+^ myeloid cells in spleens of WT mice orthotopically injected with *scr-KPC* or *shRab27a-KPC* cells, at 2 weeks post-injection. Proportion of CD45^+^ cells is indicated. Error bars indicate SEM; NS – not significant, p value: *<0.05; **<0.01; ***<0.001. Data represents 3 independent experiments.

To test the ability of Rab27a-dependent changes in systemic immune composition, we orthotopically injected *scr-KPC* and *shRab27a-KPC* cells into the pancreata of WT mice. Mice were sacrificed 2 weeks after injections, and analyzed for tumor sizes as well as the frequencies of myeloid cell populations in pancreatic tumors, spleens, livers, and lungs. We observed marked changes in the frequency of myeloid cells with Rab27a knockdown in the primary tumor (Fig. 4A). Prior studies in melanoma have linked Rab27a function with reduced metastatic burden, however, this effect was also associated with reduced primary tumor growth, complicating the analysis of metastatic outgrowth^10^. We, on the other hand, demonstrated that in pancreatic cancer, Rab27a has a role in facilitating systemic changes in immune milieu that are independent of its’ potential role in regulating primary tumor growth (Fig. 3F).

The most profound effect of Rab27a knockdown was observed in livers, where both CD11b^+^Ly6C^+^Ly6G^+^ granulocytic cells; CD11b^+^Ly6C^+^Ly6G^-^ monocytic cells and CD11b^+^Ly6C^-^Ly6G^-^ macrophage populations were significantly decreased upon knockdown of Rab27a in the primary tumor (Fig. 4B). Among the myeloid populations in spleens and lungs, monocytic CD11b^+^Ly6C^+^Ly6G^-^ and granulocytic CD11b^+^Ly6C^+^Ly6G^+^ cells were decreased, but only reduction in CD11b^+^Ly6C^-^Ly6G^-^ macrophages was found to be significant (Fig. 4C). Among the myeloid populations in the pancreas, CD11b^+^Ly6C^+^Ly6G^+^ granulocytic cells were significantly reduced, whereas there was no effect on CD11b^+^Ly6C^+^Ly6G^-^ and CD11b^+^Ly6C^-^Ly6G^-^ subsets (Fig. 4C). Overall, expression of Rab27a in the primary tumor has distinct consequences for the composition of immune milieu at the primary tumor and distant organ sites with the most significant effects seen in the liver. In addition to these observations, we noticed that, although, there was reduction in certain myeloid compartments with Rab27a knockdown, a substantial proportion of myeloid cells remained, suggesting that a secondary mechanism, such as production of GM-CSF or similar factors by the cancer cells might compensate for recruitment and expansion of specific immune subsets^22^.

We also tested if reduction in the myeloid compartment in livers upon Rab27a knock-down in primary pancreatic cells is a consequence of deficiencies in myeloid compartment of the bone marrow. Expansion of myeloid compartments in cancer is often associated with expansion in bone marrow derived cells^11^. However, consistent with previous findings in melanoma, we did not find significant differences in bone marrow-associated myeloid cells between *scr-KPC* and *shRab27a-KPC* bearing animals, suggesting that the decrease in myeloid populations in livers upon Rab27a knockdown is affecting local myeloid expansion (Supplementary Figure 3A–C)^11^. We also did not observe significant differ*ences* in functional myeloid gene expression, such as production of *VEGF, NOS2* and *ARG1*, as well as levels of MHC II upon knockdown of Rab27a, again suggesting that cancer cell-associated Rab27a might contribute largely to localized expansion of myeloid cells, rather than their functional differentiation (Supplementary Figure 4A–C and data not shown).

### **Rab27a knockdown increases early metastatic seeding**

**Figure 5:**
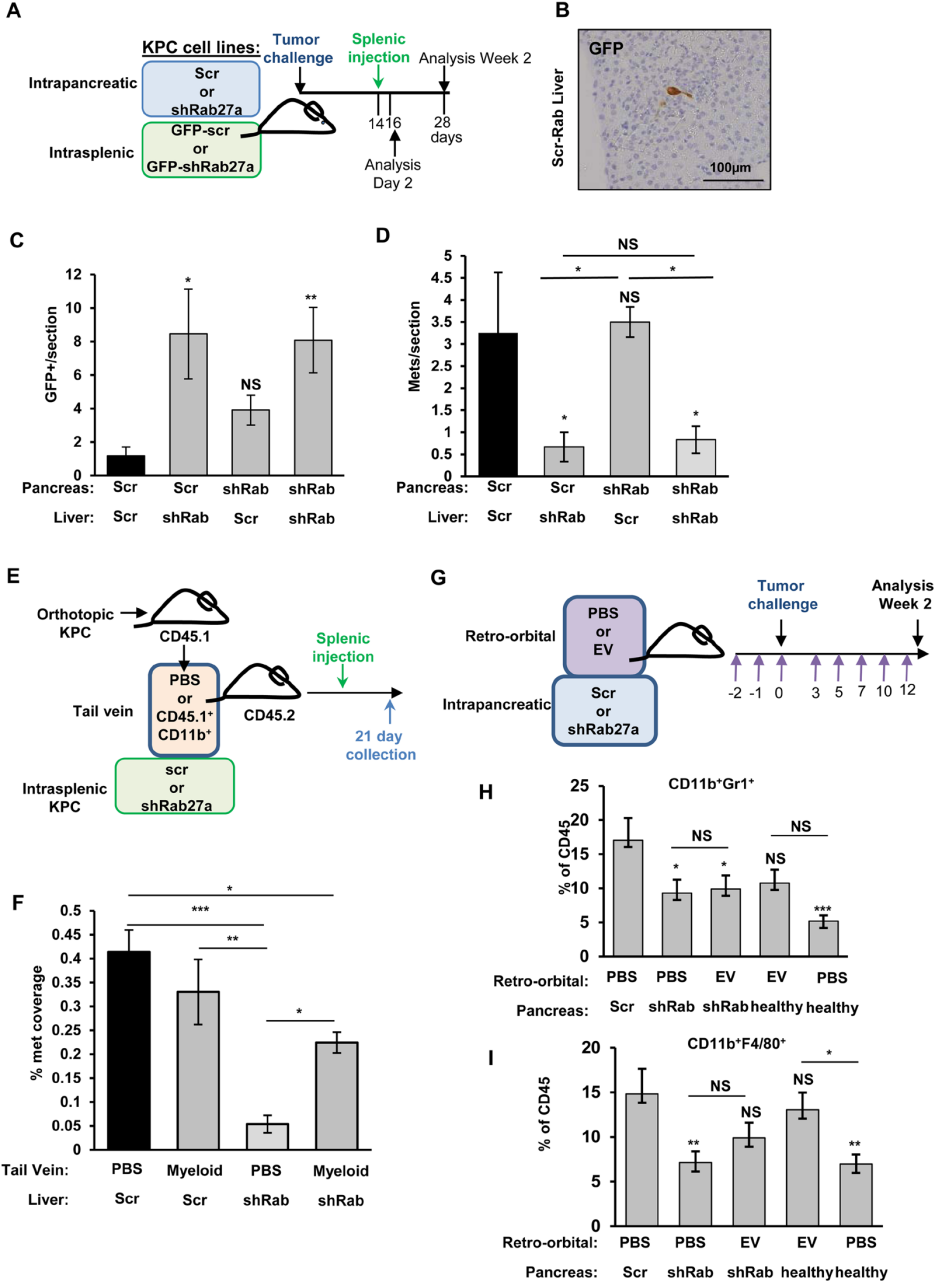
*Rab27a* knockdown increases initial metastatic seeding. **(A)** Schematic of experimental design to test the role of Rab27a in metastasis. **(B)** Representative image of *GFP-KPC* cell seeding in the liver 2 days post splenic injection as detected by GFP immunohistochemistry. **(C)** Quantification of *GFP-scr-KPC* and *GFP-shRab27a-KPC* cell seeding at 2 days post splenic injection. First label indicates the genotype of *KPC* cells orthotopically injected into the pancreas, followed by the genotype of the *KPC* cells injected into the spleen. **(D)** Quantification of *GFP-scr-KPC* and *GFP-shRab27a-KPC* cell seeding at 2 weeks post splenic injection. First label indicates the genotype of *KPC* cells orthotopically injected into the pancreas, followed by the genotype of the *KPC* cells injected into the spleen. (**E**) Schematic of experimental design to test if tumor educated myeloid cells can rescue metastatic outgrowth of shRab27a *KPC*s. (**F**) Quantification of percent of liver tissues covered by metastatic lesions from mice treated with PBS or tumor educated myeloid cells. (**G**) Schematic of experimental design to test the role of EVs in myeloid cell expansion. (**H**) Quantification of CD11b^+^ Gr1^+^ cells in WT, scr *KPC*, shRab27a mice treated with PBS or EVs. Proportion of CD45^+^ cells is indicated. (**I**) Quantification of CD11b^+^ F4/80^+^ cells in WT, scr *KPC*, shRab27a mice treated with PBS or EVs. Proportion of CD45^+^ cells is indicated. Error bars indicate SEM; statistical testing performed using one-way ANOVA with Sidak post-hoc correction for multiple comparisons where applicable: NS – not significant, p value: *<0.05; **<0.01; ***<0.001.

To understand how expression of Rab27a at the primary tumor site affects metastatic outgrowth, we have designed experiments, where *scr-KPC* or *shRab27a-KPC* cells were orthotopically injected into pancreata of WT mice, and then *GFP-scr-KPC* or *GFP-shRab27a-KPC* cells were injected intrasplenically 2 weeks post-orthotopic injection, at the time when we know that the immune niche at the distant organ sites has been established (Fig. 5A,B). We then analyzed the extent of metastatic seeding at early stages (2 days post-splenic injection) or active outgrowth stages (2 weeks-post splenic injection) by counting the number of GFP^+^ cancer cells/lesions in liver sections. Contrary to our expectations, we observed an increase in the initial seeding capacity of *GFP-shRab27a-KPC* cells regardless of whether the primary tumor was proficient or deficient in Rab27a expression (Fig. 5B, C). This effect was lost at 2 weeks post-splenic injection, as we found that livers of mice that were pre-conditioned with either *scr-KPC* or *shRab27a-KPC* tumors had significantly fewer micrometastatic (micro≤200 µm) lesions when *GFP-shRab27a-KPC* cells were injected into spleens (Fig. 5D) and no macrometastaic lesions (macro≥200 µm) were present in any group. In addition, *GFP-scr-KPC* cells generated more metastases even in the context of Rab27a deficient primary tumors, suggesting that Rab27a-mediated primary tumor-induced myeloid expansion is not required for metastatic outgrowth. (Fig. 5D).

Myeloid cells, such as liver Kupffer macrophages and bone marrow-derived monocytes, have been shown to facilitate pancreatic metastasis formation^11^. To follow up this observation, we attempted to deplete a subset of myeloid cells using anti-Gr1 antibody treatment. Anti-Gr1 treatment successfully depleted myeloid cells at the primary tumor site but was relatively ineffective in depleting the myeloid population in livers (Supplementary Figure 5A,B). This is consistent with studies by other groups, which suggest that difficulty with depletion of myeloid cells in livers of cancer-bearing mice could arise as a result of rapid repopulation by circulating cells.^23^.

To test if cancer-educated myeloid cells were sufficient to rescue metastatic outgrowth of *shRab27a-KPC* primary tumors, we adoptively transferred splenic myeloid cells from tumor-bearing CD45.1 mice (Fig. 5E). The presence of CD45.1 cells was verified at 3 weeks in the spleen as well as in liver metastases by immunohistochemistry (Supplementary Figure 5C). We observed that supplementation with myeloid cells partially rescued metastatic outgrowth *of shRab27a-KPC cells* at 3 weeks post-injection by counting both micro and macrometastases (micro≤200 µm, macro≥200 µm) (Fig. 5F). This suggests that addition of cancer-educated myeloid cells can partially compensate for deficient metastatic outgrowth in the absence of Rab27a expression in cancer cells. Partial nature of such rescue could be due to either insufficient number of transferred myeloid cells, requirement for liver-resident myeloid cell expansion as opposed to splenic and/or the possibility that additional non-myeloid-cell dependent mechanisms contribute to the successful outgrowth of Rab27a-deficient cells at metastatic sites. To understand if EVs were sufficient to modulate myeloid cell frequency, we isolated EVs from supernatants of *scr-KPC* cells. EVs or PBS control were retro-orbitally injected into WT mice or mice bearing *scr-KPC* or *shRab27a-KPC* primary tumors (Fig. 5G). WT healthy mice treated with EVs demonstrated a significant increase in intrahepatic macrophages compared to control treated mice, however the increase in Gr1^+^ cells failed to reach statistical significance (Fig. 5H,I). Overall, we did not observe changes in MHC II or CD206 marker expression (data not shown). Supplementation with EVs, however, did not rescue intrahepatic CD11b^+^F4/80^+^ or CD11b^+^Gr1^+^ cell frequencies in mice with primary *shRab27a-KPC* tumors (Fig. 5H,I). These results indicate that EVs from pancreatic cancer cells can contribute to myeloid accumulation in the healthy liver, but also suggest that non-EV-dependent mechanisms may play a role in Rab27a-mediated myeloid modulation^12^.

### **Knockdown of Rab27a results in increased expression of EMT related pathways and invasive morphology of primary tumors**

**Figure 6:**
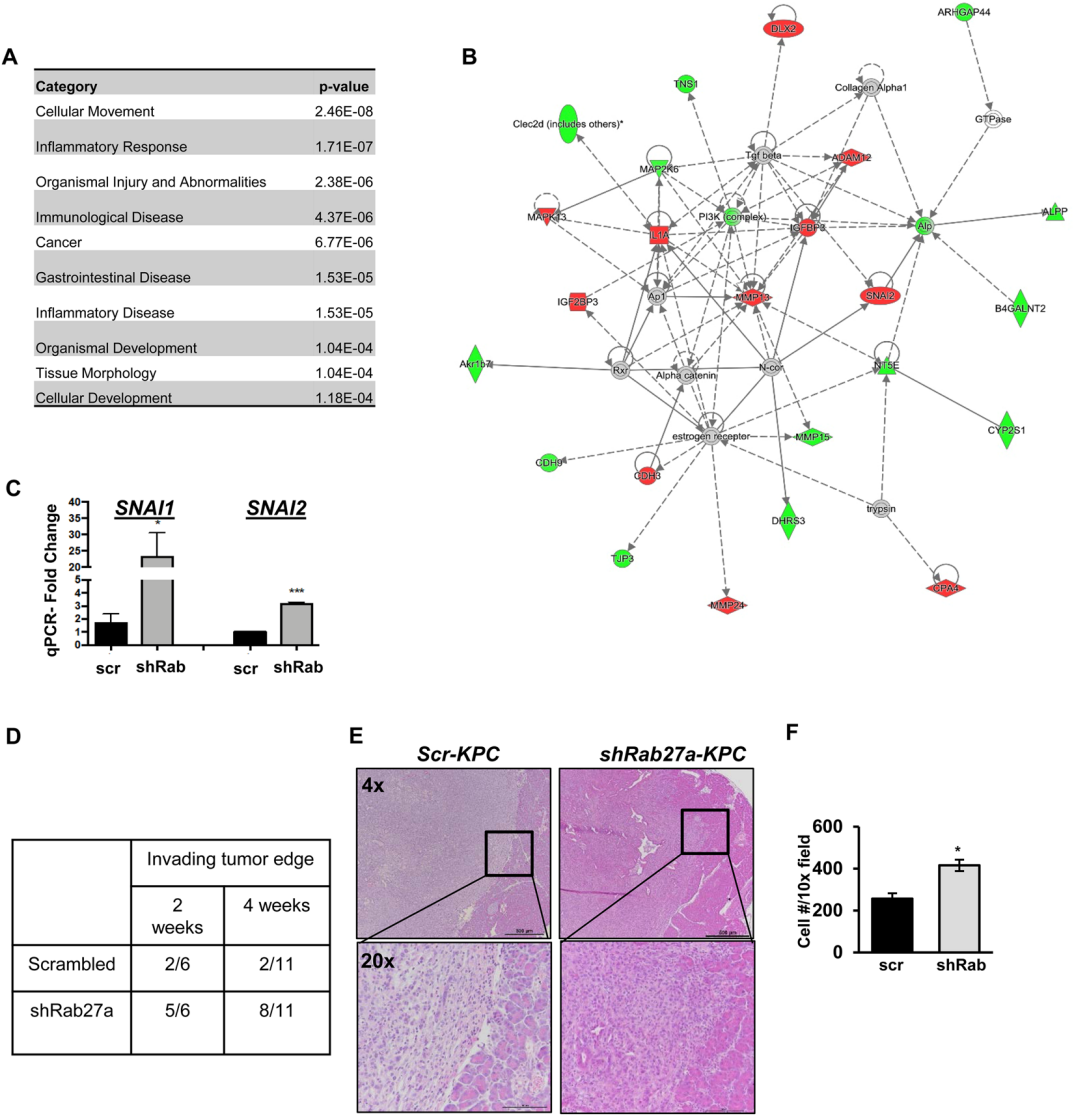
Knockdown of Rab27a results in increased expression of EMT related genes. **(A)** 10 representative diseases and functions of the 10 identified using Ingenuity Pathway Analysis of genes 2 fold up or down regulated between *shRab27a* and scrambled infected *KPC* cells. The full list can be found in Supplementary Table 1. **(B)** Example network showing altered expression of EMT related genes. A full list of identified networks can be found in Supplementary Table 2. **(C)** Table of the number of tumors with invading tumor edge for *scr*-*KPC* and *shRab27a*-*KPC* at 2 and 4 weeks. Represented as the number tumors with an invading tumor edge out of the total number evaluated in that group. **(D)** Quantitative RT-PCR of *SNAI1* and *SNAI2* genes identified as upregulated by the microarray analysis. **(E)** Representative H&E of *scr-KPC* and *shRab27a-KPC* tumors demonstrating the margin between tumor and normal tissue in *scr*-*KPC* and *shRab27a*-*KPC* at 4x and 20×. **(F)** Quantification of Transwell invasion assay of scr-*KPC* and shRab27a-*KPC* cells *in vitro*. Error bars indicate SEM; p value: *<0.05; ***<0.001. Data represents 3 independent experiments.

To understand how expression of Rab27a could modulate cell-autonomous regulation of invasion, we performed a microarray analysis of *scr-KPC* and *shRab27a-KPC* cells (both hairpin constructs). GeneSpring (Agilent) was used to analyze the microarray data. We generated a list of all genes that had a 2-fold or greater change in expression between shRab27a knockdown lines as compared to the scrambled control. To better understand the broad implications in these genes we utilized Ingenuity Pathway Analysis (IPA) (Qiagen) to identify pathways, molecular mechanisms, and biological disease states associated with the genes identified in the microarray. IPA revealed significant enrichment in diseases and functions associated with cell motility and inflammatory responses (Fig. 6A and Supplementary Tables 1 - 3). Furthermore, among the cell motility network of genes we have identified networks containing upregulation in known epithelial-to-mesenchymal transition (EMT) drivers such as Snail and Slug (Fig. 6B). We validated the significant overexpression of these genes using QPCR analysis on *scr-KPC* and *shRab27a-KPC* cells (Fig. 6C). Knockdown of *Rab27a* correlated with decrease in differentiation markers and downregulation of Trefoil Factors that are thought to inhibit EMT (Supplementary Table 4)^24–29^. Furthermore, we identified multiple metalloproteases as well as growth factors associated with remodeling of extracellular matrix and EMT conversion such as MMP13, Wnt7a, PGF and FGF18 that were significantly upregulated in our dataset, providing further support for the perturbed tumor phenotype of *shRab27a-KPC* (Supplementary Table 4)^30–38^. These findings suggested that Rab27a may suppress cell-autonomous regulation of EMT-like process in pancreatic cancer cells. In support of this idea, our analysis of primary tumors revealed that while *scr-KPC* derived tumors maintained a continuous border and did not invade surrounding pancreatic tissue, *shRab27a-KPC* tumors exhibited invasive morphology and colonized normal pancreatic tissue (Fig. 6D,E). Examination of histological sections indicated consistent predominance of lymphocytes over neutrophils in the Rab27a knockdown samples (scrambled LNR at 3.3 ± 2.9; shRab27a LNR at 3.2 ± 3.1, p value = 0.958; n = 4 samples/group). Additionally, shRab27a-KPC tumors also demonstrate disorganization of smooth muscle actin (SMA) positive regions, and loss of E-cadherin organization at the tumor margins (Supplementary Figure 6A,B). We also observed that shRab27a-KPC cells had elevated basal levels of phospho-Erk activation and were significantly more invasive *in vitro* (Fig. 6F and Supplementary Figure 6C).

Overall, these observations suggest that Rab27a may be important for suppressing some of the intrinsic abilities of cancer cells to invade tissue at early seeding stages. Furthermore, Rab27a-proficiency by cancer cells is important for their ability to colonize distant organs independent of pre-metastatic niche conditioning, potentially through alterations in EMT and/or mesenchymal-to-epithelial (MET) properties.

## Discussion

Rab27a is a Rab GTPase that functions to facilitate extracellular vesicle release from the cells^17^. Function of Rab27a in cancer has been previously linked to non-cell autonomous effects of downregulating EV production in controlling tumor growth and metastasis^10,39–43^. Rab27a is only one component of protein machinery that is important for EV biogenesis. The selection of Rab27a is motivated by several factors: it has been shown to modulate EV biogenesis in the setting of cancer^12^ and has been indirectly implicated in facilitating pre-metastatic niche formation^10^. Rab27a is overexpressed in pancreatic cancer and predicts poor survival^18^. Furthermore, high-throughput efforts aimed at finding inhibitors of EV biogenesis for therapeutic purposes are underway, and understanding the consequences of targeting such pathway(s) on a genetic level, such as by decreasing Rab27a function would provide valuable information^44^. Thus, emerging data suggests, that Rab27a could in fact have both cell-autonomous as well as non-cell-autonomous effects on tumor progression^12,45^, however, this has not been yet studied in a fully immune-competent system in order to address both aspects.

Using a combination model of experimental liver metastasis and primary tumor seeding, we demonstrated that Rab27a has both a cell-autonomous and non-cell autonomous role in modulation of the immune niche and metastatic outgrowth. Our data reinforces the notion that immune cells accumulate not only in the cancerous lesions of the pancreas but also at the sites of future metastasis, such as the liver. EVs are utilized by numerous cell types, including cancer cells. We utilized an *in vivo* mouse model in which we could alter EV release by knocking down Rab27a. Additionally, we added EVs isolated in culture back into mice with shRab27a-KPC tumors. By utilizing an *in vivo* model of pancreatic cancer, we show that primary tumors lead to expansion of myeloid subsets in distant sites such as the spleen, liver, and lung. This systemic regulation can promote seeding of metastasis-incompetent pancreatic cancer cell lines in the liver. Expression of Rab27a at the site of the primary tumor mediates not only changes in EV production, but regulates expression of multiple secreted factors associated with EMT processes and tumor remodeling. Consistent with this idea, downregulation of Rab27a led to decreases in myeloid subsets both at the primary tumor site and distant sites such as spleen, lung, and liver. We noticed that depending on the organ site, the nature of immune regulation by Rab27a was distinct: although the type of cells differed – both macrophages and MDSC were downregulated in liver, while MDSCs alone were decreased in the primary pancreatic tumor and macrophages alone were decreased in the spleen and lung. This suggests that additional systemic sources, such as remaining GM-CSF may potentially contribute to recruitment of these cells. In addition to previously reported ability of primary pancreatic cancer to increase macrophage frequencies in livers, we report that Rab27a may also regulate different subtypes of myeloid cells in livers. However, in lungs and spleens, only macrophages are affected, whereas we also noticed that knockdown of Rab27a affected recruitment of Gr1^+^ cells to primary pancreatic lesions. Therefore, it seems that either different populations of immune cells at targeted organ sites are regulated by Rab27a proficient cancer cells; or there might be distinct types of systemic cues that are regulated by Rab27a that confer organ specificity.

To understand how expression of Rab27a affects tumor progression in an immune-competent model, we pre-conditioned mice by implanting primary tumors for two weeks. Surpisingly, direct delivery of Rab27a knockdown cells to livers of pre-conditioned mice revealed an increase in the short-term seeding ability. We found that downregulation of Rab27a perturbed expression of genes related to cell motility and increased an EMT gene signature in the cancer cells. This was consistent with results reported in hepatocellular carcinoma cells and could be a result of enhanced internal signaling by the components of unreleased EVs^45^. Concordant with this, primary tumors were much more invasive with downregulation of Rab27a. In contrast, primary tumors with downregulated Rab27a expression could not support long-term seeding of metastatic nodules. This could reflect overall changes in inflammatory modulators produced by Rab27a knockdown cells. Since knockdown of Rab27a resulted in increased EMT, one possibility is that these cells may have impaired ability to undergo mesenchymal-to-epithelial transition (MET), which is characterized by reversion to the epithelial phenotype and has been implicated in potentiating successful metastatic outgrowth^46^.

Overall, our work identified that the GTPase Rab27a functions in both a cell-autonomous as well as a non-cell-autonomous fashion to affect tumor progression. Disrupted EV biogenesis in cancer cells by virtue of downregulation of Rab27a may have a cell-autonomous role in promoting EMT processes that promote primary tumor invasion and increase the initial seeding rate of metastases, whereas systemic factors regulated by Rab27a may be necessary to re-program pre-metastatic niche and facilitate long-term survival of metastatic lesions.

## Materials and Methods

All experiments were performed in accordance with relevant guidelines and regulations.

### Mice

All mouse protocols were reviewed and approved by the Institutional Animal Care and Use Committee (IACUC) of the University of North Carolina at Chapel Hill. All animals were maintained in specific pathogen-free facilities. Six to eight week-old wild-type C57Bl/6 mice were purchased from the Charles River Laboratories (stock #027). Both males and female mice were used for orthotopic and splenic injections of PDA cells. For orthotopic injection of cancer cells into the pancreas, mice were anesthetized using a Ketamine (100 mg/kg)/Xylazine (10 mg/kg) cocktail administered via intraperitoneal injection. Depth of anesthesia was confirmed by verifying the absence of a toe pinch response. A small incision was made in the left flank and 100,000 KPC cells in ice-cold PBS mixed at a 1:1 dilution with Matrigel (#354234, Corning) in a volume of 50ul was injected into the tail of the pancreas using a 28-gauge needle. The incision was closed in two layers, with running 5-0 Vicryl Rapide sutures (Ethicon) for the body wall, and interrupted 5-0 Prolene sutures (Ethicon) for the skin. Pain was managed using subcutaneous administration of Buprenorphine (0.1 mg/kg). For splenic injections, 500,000 KPC cells in 100ul of ice cold PBS were injected into the spleen using a 28-guage needle. Mice receiving a splenic injection after a primary orthotopic injection were injected with 200,000 KPC cells. Mice were euthanized by carbon dioxide-induced narcosis at two weeks or four weeks post injection of cancer cells. Tumors were measured using digital calipers and processed for histology and flow cytometry. Livers, lungs, and spleens were collected and processed for histology and flow cytometry.

### Pancreatic Cancer Cell lines

The murine PDA cell line 4662 was derived from primary pancreatic tumors *LSL-Kras*^*G12D/+*^*; LSL-Trp53*^*R172H/+*^*; p48*^*Cre/+*^ (*KPC*) mice on C57Bl6/J background, as reported in Bayne et al., and was a kind gift from Dr. Vonderheide^47^. GFP-labeled PDA lines were generated as previously described^22^. Short-hairpin RNAs (TRCN0000100577 and TRCN0000100578) available from the Broad Institute were utilized to knockdown the expression of Rab27a. To generate lentiviral particles, HEK-293T cells were co-transfected with the vector, the packaging construct (pHR-CMV-dR8.2), and the envelope plasmid (pCMV-VSVG). Viral stocks were collected, filtered through a 0.45μm syringe filter, and concentrated using 100MWCO Amicon Ultra centrifugal filters (Millipore). A multiplicity of infection of 15 was used for lentiviral infection of WT- or *Kras*^*G12D*^-PDEC in the presence of 10 µg/ml Polybrene (Chemicon). Rab27a knockdown was verified by qPCR for *Rab27a* (forward 5′ AAGGGATAGAGCACAGCGAG 3′ and reverse 5′ TGCAGTGTAGCGTCCTTAGC 3′) and *Gapdh* (forward 5′ CACGGCAAATTCAACGGCACAGTC and 3′ reverse 5′ ACCCGTTTGGCTCCACCCTTCA 3′). Cells lines were maintained at 37^0^C, 5% CO_2_.

### Extracellular vesicle collection

*KPC* cells were seeded in 10 cm dishes and allowed to reach 70% confluency and then the media was changed to serum-free DMEM and incubated for an additional 24–48 hours. Supernatant was collected, spun briefly, filtered through a 0.22um filter to remove cell debris, and incubated with 40% PEG overnight at a ratio of 1:4 (PEG to supernatant). Supernatant and PEG solutions were then spun at 3500 rpm for 30 minutes. Supernatant was discarded and EVs were resuspended in PBS and quantified on the NanoSight. For mice treated with extracellular vesicles, the vesicles were quantitated by a Pierce BCA assay (Thermo Fisher). Mice were treated with 5 µg of extracellular vesicles diluted in PBS or PBS alone as a control^11^ by retro-orbital injection. Mice were treated with extracellular vesicles two days prior to orthotopic implantation, the day of implantation, and then 3 times per week until harvest.

### Proliferation and viability assays

For *KPC* growth assays, cells were seeded at a density of 1,000 cells/well in a 96-well tissue culture plate. At indicated time points, the cell culture medium was aspirated and the wells were washed with RPMI (without phenol red, BioWhittaker). 0.5 mg/ml 3-[4,5-dimethylthiazol-2-yl]-2,5-diphenyl tetrazolium bromide (MTT; Sigma-Aldrich) was added to the wells for 2 hours at 37 °C. The reagent was aspirated and 100 μl of DMSO was added to each well for 20 minutes at room temperature. Plates were read at an absorbance of 570 nm with background subtraction at 690 nm using a Synergy 2 microplate reader (BioTek). For the day 0 time point, the cells were treated with the MTT reagent 12-18hrs after plating.

### Invasion assay

Transwells (8 µm) (VWR) were coated with 2 µg of Matrigel per well and allowed to dry overnight. 100,000 *KPC* cells were seeded on top of the coated-chambers in 300ul serum free DMEM (Gibco). The lower chambers contained 800ul of complete media. Inserts were fixed in 4% paraformaldehyde for 10 minutes at room temperature. The cells remaining in the top chamber were scraped by cotton swabs, while the invading cells were stained with crystal violet (Sigma) for 5 minutes at room temperature. Each Transwell was imaged at 10x magnification with 3 individual images counted per well. The assay was run in triplicate.

### Lymphocyte isolation

Single cell suspensions were prepared from tumors, spleens, livers, and lungs. Spleens were mechanically disrupted and resuspended in 1% FBS/PBS. Pancreatic tumors were minced and digested with 1.25 mg/ml collagenase IV (Cat. No.LS004188, Worthington), 1 mg/ml Hyaluronidase (Cat. No. LS 002592, Worthington), 0.1% Trypsin Inhibitor from soybean (Cat. No. T9128, Sigma), and 100ug/ml DNase I (Cat. No. LS002007, Worthington) in RPMI for 30 minutes at 37 °C. Livers were minced and digested in 1 mg/ml collagenase IV (Cat. No.LS004188, Worthington) and 100ug/ml DNase I (Cat. No. LS002007, Worthington) in RPMI for 30 minutes at 37 °C. Lungs were perfused, minced, and digested in 0.5 mg/ml Collagenase IV (Cat. No.LS004188, Worthington) and 100ug/ml DNase I (Cat. No. LS002007, Worthington) in RPMI for 45 minutes at 37 °C. Cell suspensions were passed through a 70 µm strainer and resuspended in 1%FBS/PBS. 1x RBC lysis buffer (eBioscience, #00-4333-57) was used according to manufacturers instructions. Livers were further enriched for lymphocytes by OptiPrep (Sigma) density gradient centrifugation. Tumor-infiltating leukocytes were enriched using CD45-microbeads (Miltenyi, #130-052-301). MDSCs were isolated using the MDSC isolation kit (Miltenyi, #130-094-538). Once isolated, cells were washed and blocked with an α-CD16/CD32 antibody (BD Biosciences, Clone 2.4G2) for 5 minutes on ice and then stained with labeled antibodies against surface markers for 30 minutes in 1% FBS/PBS. Cells were also stained with Live/Dead Aqua (Thermo Fisher, #L34966) in PBS to determine viability and fixed in 2% PFA. The following mAbs directed against mouse antigens were used for flow cytometry: α-Gr1-FITC (Clone: Rb6-8C5, Biolegend), or α-Ly6C-AF488 (Clone: HK1.4, Biolegend) and α-Ly6G-AF700 (Clone: 1A8, Biolegend), α-F4/80-PerCP-Cy5.5 (Clone: BM8, Biolegend), α-CD45-Pacific Blue (Clone: 104, Biolegend), α-CD11b-APC (Clone: M1/70, Biolegend). All samples were acquired on LSR II or LSRII Fortessa (BD Bioscience) at the UNC Flow Cytometry Core Facility and analyzed using FlowJo (Treestar, Inc.).

### Immunohistochemistry

Mouse tumors, spleens, livers and lungs were fixed in 10% phosphate-buffered formalin (Fisher Scientific) for 24-48 hours and embedded in paraffin at the UNC Animal Histopathology and Laboratory Medicine Core. Six micron sections were deparaffinized and rehydrated. Endogenous peroxidase activity was quenched using a solution of 1% hydrogen peroxide (30% hydrogen peroxide stock, Sigma) in methanol at room temperature for 10 minutes. Antigen retrieval was done in 10 mM sodium citrate/0.05% Tween-20 (pH 6) for 15 minutes in a microwave oven for all antibodies other than Gr1. For antigen retrieval for Gr1 slides were treated with pronase (0.05 mg/ml) for 10 minutes at 37 °C. Blocking was performed for 1 hour at room temperature, in 10% goat serum/10 mM Tris-HCL/0.1 M magnesium chloride/1% BSA/0.5% Tween-20. Sections were incubated with primary antibodies overnight at 4 °C in humidified chambers. Secondary biotinylated goat-anti-rabbit or goat-anti-rat antibody (Vector Laboratories) was diluted in 2% BSA/PBS and incubated for 1 hour at room temperature. Tertiary ABC solution was prepared according to manufacturer’s instructions (Vectastain ABC kit, Vector Laboratories) and was incubated for 45 minutes at room temperature. Stain was developed using 3,3′-diaminobenzidine tetrahydrochloride kit (DAB peroxidase substrate kit, Vector Laboratories). Slides were counterstained with Harris hematoxylin (Sigma), dehydrated and mounted with DPX mounting media (Sigma). The following antibodies were used: α-GFP (Cell Signaling Technologies, 1:200), α-CD45 (BD Biosciences, 1:200), and α-Gr1 (Biolegend 1:100). Livers were cut at sequential depths. Specifically, sections were collected by advancing the block by 25 microns and before collecting another set of sections. This was done for at least 4 steps for each liver. Multiple liver sections were stained and counted for each time point. Early liver seeding was counted by scanning each of the liver sections at 20x magnification and counting the total number of GFP-positive cells for the section. Metastatic out growth was counted by scanning the H&E stained sections at multiple depths at 10×.

### Immunofluorescence

Mouse tumors, spleens, livers, and lungs were fixed in 10% phosphate-buffered formalin (Fisher Scientific) for 24–48 hours and embedded in paraffin at the UNC Animal Histopathology and Laboratory Medicine Core. Six micron sections were deparaffinized and rehydrated. Antigen retrieval was done in 10 mM sodium citrate/0.05% Tween-20 (pH 6.1) for 15 minutes in a microwave oven. Blocking was performed for 1 hour at room temperature, in 10% chicken serum/10 mM Tris-HCL/0.1 M magnesium chloride/1% BSA/0.5% Tween-20. Sections were incubated with primary antibodies overnight at 4 °C in humidified chambers. Sections were stained with chicken anti-rabbit alexa fluor 488 and chicken anti-rat alexa fluor 594 (Life Technologies) secondary antibodies diluted in 2% BSA/PBS and incubated for 1 hour at room temperature. Additional staining antibodies were incubated for 1 hr followed by relevant secondary for one hour at room temperature. Nuclei were stained with DAPI (Molecular Probes) and slides were mounted with Prolong Gold Anti-fade mountant (Life Technologies) and coverslipped. The following primary antibodies were used for immunofluorescence staining: α-phospho-histone 3 (Millipore, 1:150), α-alpha-smooth muscle actin (Abcam, 1:200), α-CK8 (DSHB, troma-1, 1:100), and α-E-cadherin (Abcam, 1:200).

### **Adoptive transfer of CD11b**^**+**^**myeloid cells**

CD45.1 mice were orthotopically implanted with 150,000 *KPC* cells. Approximately 2 weeks post-implantation, the spleens from the CD45.1 mice were collected and CD11b^+^ cells were isolated using a CD11b positive-selection kit (StemCell Technologies, #18103). Isolated CD11b^+^ cells were counted and resuspended in PBS. Approximately 4 × 10^6^ CD11b^+^ cells or equal volumes of PBS were injected via the tail vein into CD45.2 C57Bl/6 J mice. The following day the CD45.2 mice underwent a splenic injection of 500,000 *scr-KPC* or *shRab27a-KPC* cells. Mice were collected at 3 weeks post-splenic injection.

### Microarray methods

Microarray samples were prepared and run by the UNC Functional Genomics Core. In short, Total RNA (250 ng) was used to synthesize fragmented and labeled sense-strand cDNA and hybridized onto Affymetrix arrays. The Affymetrix GeneChip® WT PLUS Reagent Kit Manual was followed to prepare the samples. Briefly, the GeneChip® WT PLUS Reagent Kit (Affymetrix) was used to generate sense-strand cDNA from total RNA. Following synthesis of sense-strand cDNA, the cDNA was fragmented and labeled with the kit. Fragmented and labeled cDNA was used to prepare a hybridization cocktail with the Affymetrix GeneTitan Hybridization Wash and Stain Kit for WT Arrays. Hybridization, washing, staining and scanning of the Affymtrix peg plate arrays was carried out using the Affymetrix GeneTitan MC Instrument. GeneChip Command Console Software (AGCC) was used for GeneTitan Instrument control. Affymetrix Expression Console Software was used for basic data analysis and quality control. Microarray gene expression data can be found at NIH Gene Expression Omnibus (GEO) repository under accession #GSE148439.

### Quantitative PCR (qPCR) analysis

RNA was purified from cells using the RNeasy mini kit (Qiagen, 74134). RNA was normalized to 1 µg of total RNA and cDNA was generated using the Maxima First Strand cDNA Synthesis Kit (Thermo Scientific, K1671). QPCR analysis was performed using PerfeCTa SYBR Green SuperMix (Quanta Biosciences) and the Applied Biosystems (ABS) 7500 real-time PCR system. Results were normalized to the expression of *GAPDH* and then calculated using ΔΔCT. All samples were run in triplicate. *SNAI1* (forward 5′ CTGGTGAGAAGCCATTCTCCT 3′ and reverse 5′ CCTGGCACTGGTATCTCTTCA 3′) SNAI2 (forward 5′ TGGTCAAGAAACATTTCAACGCC 3′ and reverse 5′ GGTGAGGATCTCTGGTTTTGGTA 3′) primers were used for PCR amplification.

### Statistical analysis

To obtain sufficient statistical power, at least 5-12 mice were used in each group, and the experiments were repeated a minimum of 3 times to validate reproducibility. Group means were compared with student’s t-test or ANOVA with a Sidak post-hoc test. Statistical analysis were performed using GraphPad Prism software. Data are presented as mean + s.e.m. A p-value of less than 0.05 was considered statistically significant.

## Supplementary information

Supplementary Figures.

Supplementary Table 1.

Supplementary Table 2.

Supplementary Table 3.

Supplementary Table 4.
