# Supplementary material for: Rab27a plays a dual role in metastatic propensity of pancreatic cancer
Source: Sci Rep. 2020 Apr 30;10:7390. doi: 10.1038/s41598-020-64248-1 (PMC7193593; doi:10.1038/s41598-020-64248-1)
Supplement: Supplementary file 1 — Supplementary Figures. [file 41598_2020_64248_MOESM1_ESM.pdf]

Supplementary Materials.

Rab27a plays a dual role in metastatic propensity of pancreatic cancer.

Nancy Kren, Daniel Michaud, Sukriti Bagchi, Kevin Greene, Yuliya Pylayeva-Gupta

Supplementary Figure 1

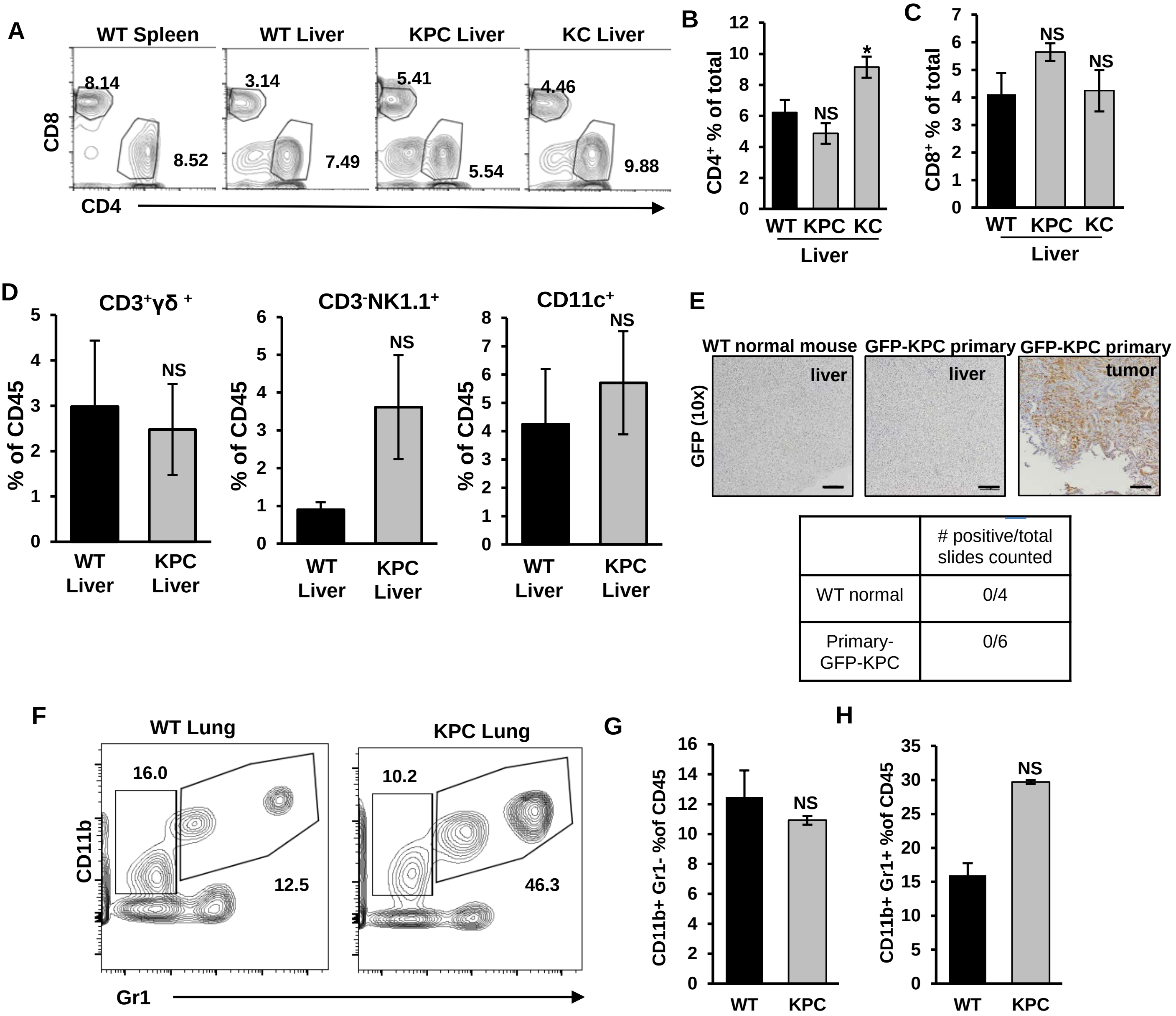

	# positive/total slides counted
WT normal	0/4
Primary-GFP-KPC	0/6

Supplementary Figure 1. Changes in immune microenvironment of distant organs in response to PDA.

(A) Representative flow cytometry analysis of $CD3^+$ $CD4^+$ and $CD8^+$ in WT, *KPC* and *KC* livers. Proportion of total cells is indicated.
(B) Quantification of $CD4^+$ cells in WT, *KPC* and *KC* livers. Proportion of total cells is indicated.
(C) Quantification of $CD8^+$ cells in WT, *KPC*, and *KC* livers. Proportion of total cells is indicated.
(D) Quantification of $\gamma\delta^+$ T cells, NK cells and $CD11c^+$ cells in WT and KPC livers. Proportion of $CD45^+$ cells is indicated.
(E) IHC and quantification of GFP in WT normal liver, or in the liver of mice with a primary GFP-KPC tumor. Number of liver sections positive for GFP cells out of the total number of sections stained is shown. Scale bar, 100µm.
(F) Representative flow cytometry analysis of $CD45^+CD11b^+Gr1^+$ myeloid populations in lungs from WT normal mouse or a *KPC* mouse. Proportion of $CD45^+$ cells is indicated.
(G) Quantification of $CD45^+CD11b^+Gr1^-$ macrophages from the lungs of WT and KPC mice. Proportion of $CD45^+$ cells is indicated.
(H) Quantification of $CD11b^+Gr1^+$ myeloid cells from the lungs of WT and *KPC* mice. Proportion of $CD45^+$ cells is indicated.

Error bars indicate SEM; NS – not significant. p value: * $p< 0.05$

Supplementary Figure 2

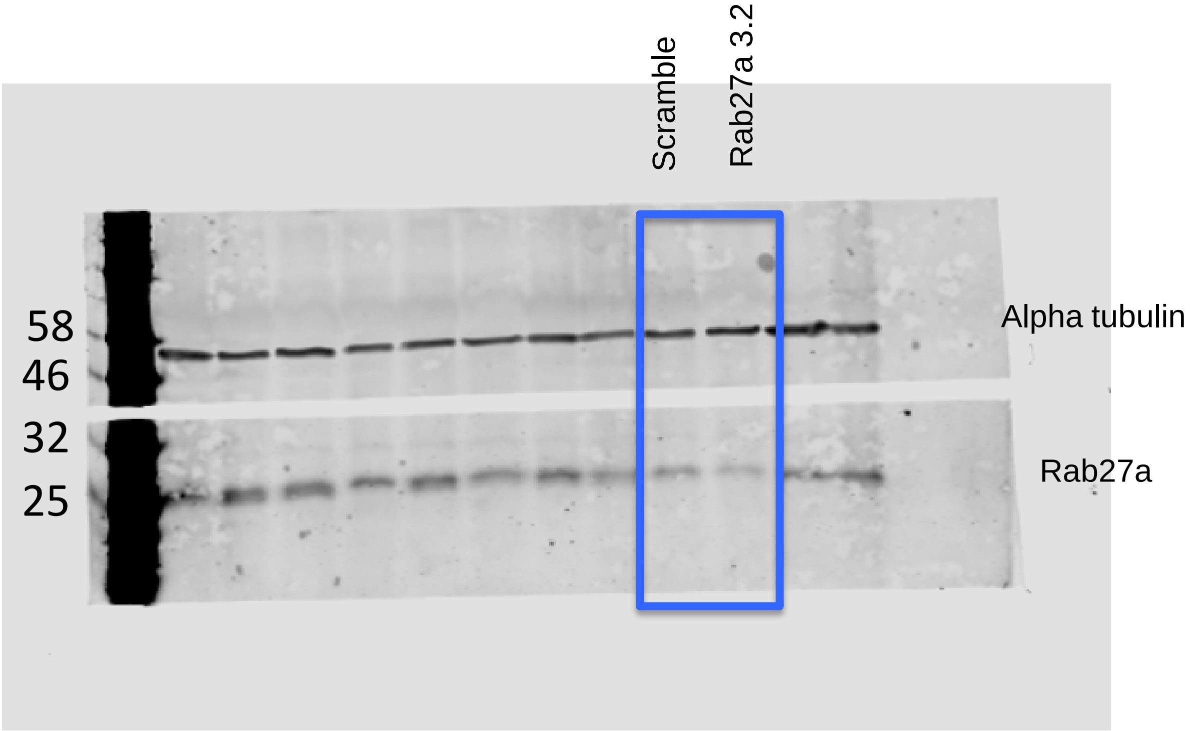

Supplementary Figure 2. Levels of Rab27a protein in KPC cells were assessed by Western blotting.

Supplementary Figure 3

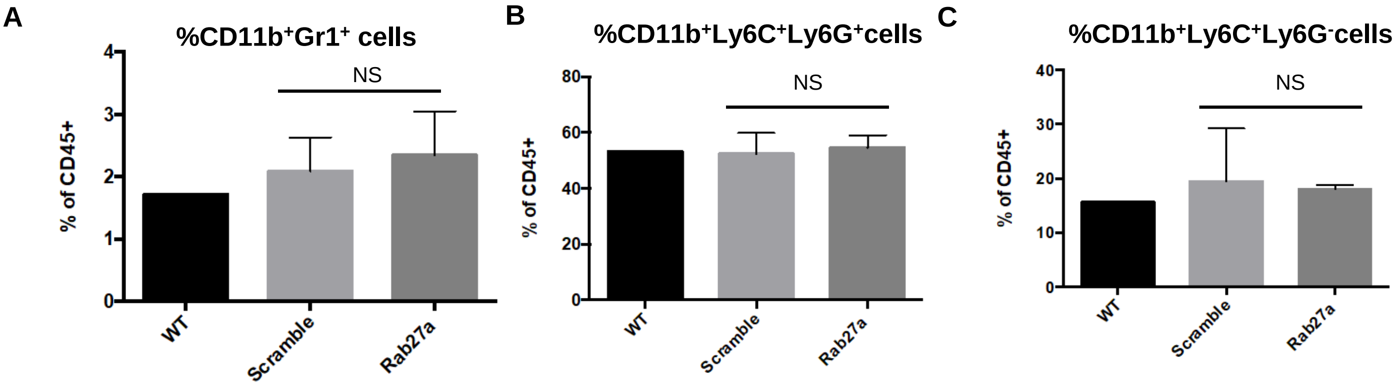

Supplementary Figure 3. Reduction in Rab27a expression in the primary tumor cells does not affect frequency of myeloid cells in bone marrow.

(A) Quantification of CD11b$^+$Gr1+ myeloid cells from the bone marrow of naïve WT mice or mice orthotopically injected with *scr-KPC* or *shRab27a-KPC*. Proportion of CD45$^+$ cells is indicated.

(B) Quantification of CD11b$^+$Ly6C$^+$Ly6G$^+$ cells from the bone marrow of naïve WT mice or mice orthotopically injected with *scr-KPC* or *shRab27a-KPC*. Proportion of CD45$^+$ cells is indicated.

(C) Quantification of CD11b$^+$Ly6C$^+$Ly6G$^-$ cells from the bone marrow of naïve WT mice or mice orthotopically injected with *scr-KPC* or *shRab27a-KPC*. Proportion of CD45$^+$ cells is indicated.

Error bars indicate SEM; NS – not significant.

Supplementary Figure 4

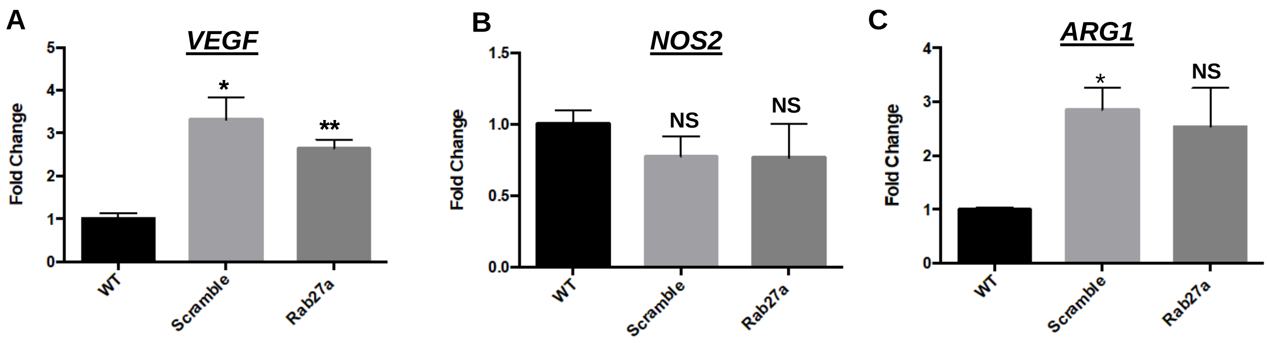

Supplementary Figure 4. Reduction in Rab27a expression in the primary tumor cells does not affect expression of functional proteins in myeloid cells.

(A) QPCR of *VEGF* from CD11b$^+$Ly6C$^+$ cells isolated from spleens of naïve WT mice or mice orthotopically injected with *scr-KPC* or *shRab27a-KPC*.

(B) QPCR of NOS2 from CD11b$^+$ Ly6C$^+$ cells isolated from spleens of naïve WT mice or mice orthotopically injected with *scr-KPC* or *shRab27a-KPC*.

(C) QPCR of Arg1 from CD11b$^+$ Ly6C$^+$ cells isolated from spleens of naïve WT mice or mice orthotopically injected with *scr-KPC* or *shRab27a-KPC*.

Error bars indicate SEM; NS – not significant. p value: * $p < 0.05$, ** $p < 0.01$

Supplementary Figure 5

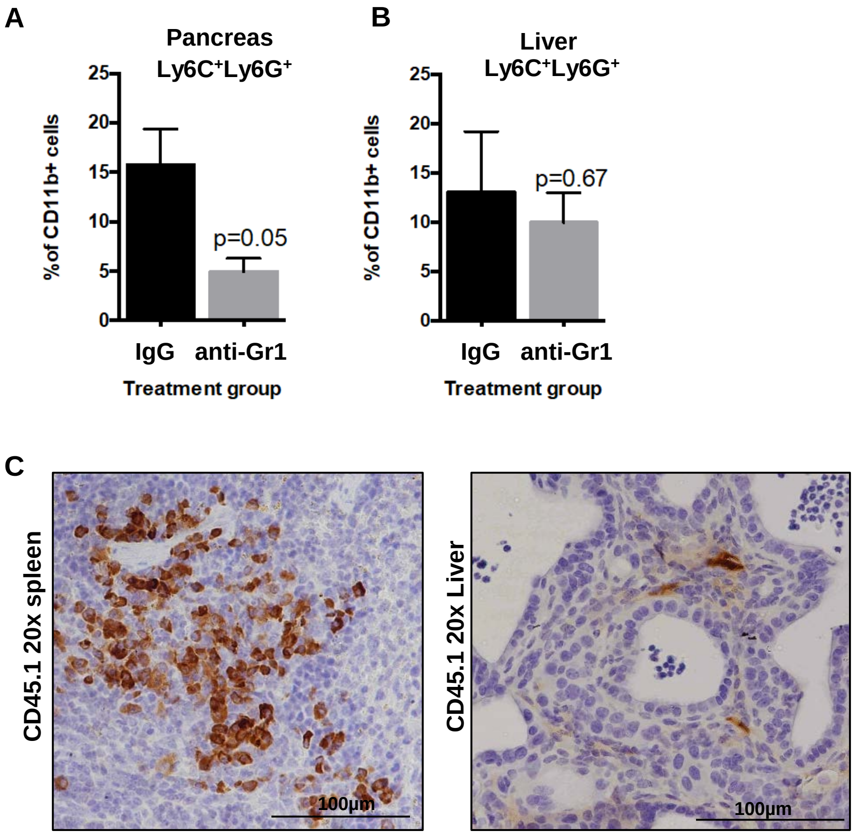

Supplementary Figure 5. Role of myeloid cells in metastatic potential of pancreatic cancer.

(A) Mice were injected with KPC into the pancreas and *KPC-GFP* into the spleen. Mice were treated with 200µg of anti-Gr1 (RB6-8C5) 3 times a week for 2 weeks. Tissues were collected at 2 weeks and lymphocytes isolated. Frequency of $CD11b^+Gr1^+$ cells is the pancreas as assessed by flow cytometry are indicated.

(B) Frequency of $CD11b^+Gr1^+$ cells is livers as assessed by flow cytometry are indicated.

(C) CD45.1 staining in Myeloid enriched mice in the spleen (20x) and a liver metastasis (20x) at 22 days post tail vein injection. Scale bar, 100 µm.

Error bars indicate SEM; NS – not significant. p value: * p< 0.05, ** p< 0.01, ***p<0.001

Supplementary Figure 6

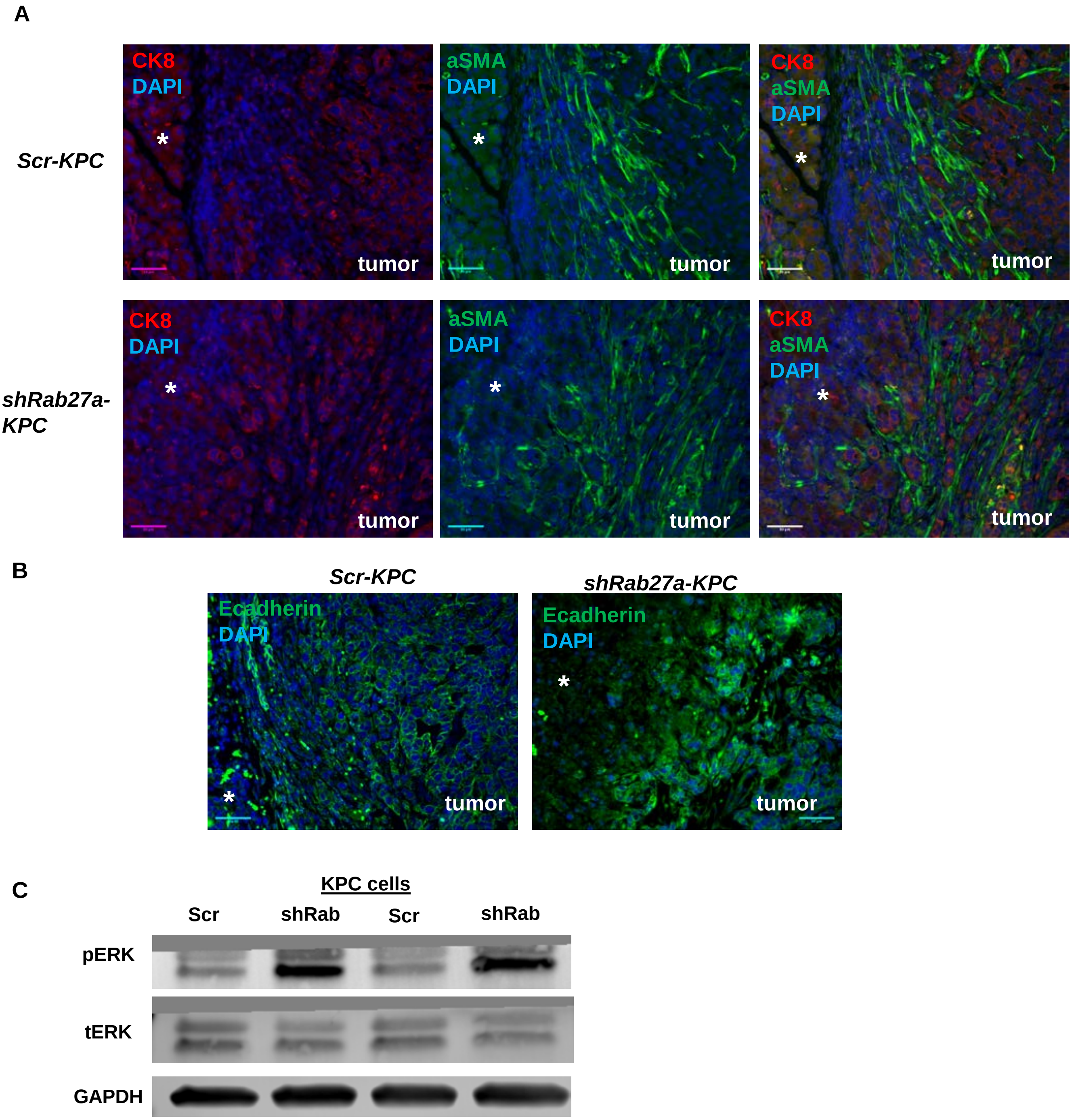

Supplementary Figure 6. Knockdown of Rab27a reveals cell autonomous changes in KPC cells.

(A) Immunofluorescent staining of scr-KPC and shRab27a-KPC primary tumors with CK8 and αSMA, at the boundary with normal acinar tissue. Location of normal acinar tissue is marked with an asterisk. Scale bars, 50 µm.

(B) Western blot demonstrating increased phospho-Erk in *shRab27a-KPC* cells.

(C) Immunofluorescent staining of scr-KPC and shRab27a-KPC primary tumors with E-cadherin, at the boundary with normal acinar tissue. Direction of normal acinar tissue is marked with an asterisk. Scale bars, 50 µm.
